# Beneficial Effect of a Selective Adenosine A_2A_ Receptor Antagonist in the APPswe/PS1dE9 Mouse Model of Alzheimer’s Disease

**DOI:** 10.3389/fnmol.2018.00235

**Published:** 2018-07-12

**Authors:** Emilie Faivre, Joana E. Coelho, Katja Zornbach, Enas Malik, Younis Baqi, Marion Schneider, Lucrezia Cellai, Kevin Carvalho, Shéhérazade Sebda, Martin Figeac, Sabiha Eddarkaoui, Raphaëlle Caillierez, Yijuang Chern, Michael Heneka, Nicolas Sergeant, Christa E. Müller, Annett Halle, Luc Buée, Luisa V. Lopes, David Blum

**Affiliations:** ^1^Université de Lille, Inserm, CHU-Lille, LabEx DISTALZ, Jean-Pierre Aubert Research Centre UMR-S1172, Alzheimer & Tauopathies, Lille, France; ^2^Instituto de Medicina Molecular, Faculdade de Medicina de Lisboa, Universidade de Lisboa, Lisbon, Portugal; ^3^Center of Advanced European Studies and Research, Bonn, Germany; ^4^PharmaCenter Bonn, Pharmaceutical Institute, Pharmaceutical Chemistry I, University of Bonn, Bonn, Germany; ^5^Department of Chemistry, Faculty of Science, Sultan Qaboos University, Muscat, Oman; ^6^Plateau de Génomique Fonctionnelle et Structurale, CHU Lille, University of Lille, Lille, France; ^7^Institute of Biomedical Sciences, Academia Sinica, Taipei, Taiwan; ^8^German Center for Neurodegenerative Diseases (DZNE), Bonn, Germany; ^9^Department of Neurodegenerative Diseases and Geropsychiatry/Neurology, University of Bonn Medical Center, Bonn, Germany

**Keywords:** Alzheimer’s disease, amyloid, adenosine receptor, A_2A_, memory

## Abstract

Consumption of caffeine, a non-selective adenosine A_2A_ receptor (A_2A_R) antagonist, reduces the risk of developing Alzheimer’s disease (AD) and mitigates both amyloid and Tau lesions in transgenic mouse models of the disease. While short-term treatment with A_2A_R antagonists have been shown to alleviate cognitive deficits in mouse models of amyloidogenesis, impact of a chronic and long-term treatment on the development of amyloid burden, associated neuroinflammation and memory deficits has never been assessed. In the present study, we have evaluated the effect of a 6-month treatment of APPsw/PS1dE9 mice with the potent and selective A_2A_R antagonist MSX-3 from 3 to 9-10 months of age. At completion of the treatment, we found that the MSX-3 treatment prevented the development of memory deficits in APP/PS1dE9 mice, without significantly altering hippocampal and cortical gene expressions. Interestingly, MSX-3 treatment led to a significant decrease of Aβ1-42 levels in the cortex of APP/PS1dE9 animals, while Aβ1-40 increased, thereby strongly affecting the Aβ1-42/Aβ1-40 ratio. Together, these data support the idea that A_2A_R blockade is of therapeutic value for AD.

## Introduction

Alzheimer’s disease (AD) is the most common neurodegenerative disorder in the elderly. AD is characterized by a progressive cognitive decline and neuropathologically defined by two hallmarks: extracellular deposits constisting of aggregated β-amyloid (Aβ) peptides and intraneuronal fibrillar aggregates of hyper- and abnormally phosphorylated Tau proteins (Masters et al., 1985; Duyckaerts et al., 2015). AD is dependent on various genetic and environmental factors (Reitz et al., 2011; Cuyvers and Sleegers, 2016). Among protective factors, several epidemiological studies have reported an inverse relation between caffeine intake, age-related cognitive impairments and the risk to develop AD later in life (for reviews see Flaten et al., 2014; Cunha, 2016). In accordance, we and others have shown that caffeine is beneficial towards memory impairments and pathology in transgenic mouse models of AD (Arendash et al., 2006, 2009; Cao et al., 2009; Laurent et al., 2014).

Beneficial effects of caffeine have been ascribed to its ability to block adenosine A_2A_ receptors (A_2A_Rs), a G protein-coupled receptor whose endogenous ligand is adenosine (Cunha, 2016). In line with a role of A_2A_Rs in AD, an association between a polymorphism of the ADORA2A gene with hippocampal volume in mild cognitive impairment and AD has been recently reported (Horgusluoglu-Moloch et al., 2017). Like caffeine, pharmacological and genetic A_2A_R blockade was found to reduce hippocampal pathology, neuroinflammation and memory deficits in a model of AD-like Tau pathology (Laurent et al., 2016). A_2A_R blockade or deletion was also found to counteract synaptotoxicity and memory deficits acutely induced by Aβ peptides (Dall’lgna et al., 2003, 2007; Canas et al., 2009). In line, recent data emphasize in transgenic models of amyloidogenesis (APP/PS1dE9 and hAPP-J20), that short-term treatments (from 1 to 3 weeks) with selective A_2A_R antagonists (SCH58260 i.p. or KW6002 p.o.) revert memory alterations (Viana da Silva et al., 2016; Orr et al., 2018; Silva et al., 2018). However, the impact of a long-term and chronic A_2A_R blockade on the development of amyloid pathology and associated memory impairment has not been investigated yet.

In the present study, we explored the outcomes of a chronic pharmacological blockade of A_2A_R in the APPswe/PS1dE9 transgenic mouse model, using the selective water-soluble antagonist MSX-3. Our data demonstrate that chronic MSX-3 treatment delivered from 3 to 9-10 months of age in APPsw/PS1dE9 mice improves spatial memory deficits and moderately reduces cortical amyloid load. These data support the notion that targeting A_2A_Rs is of therapeutic interest for AD.

## Materials and Methods

### Animals

In this study, we used heterozygous male APPswe/PS1dE9 (herein referred to as APP/PS1, C57Bl6/J background; Jankowsky et al., 2001) and littermates controls. All animals were maintained in standard cages under conventional laboratory conditions (12 h/12 h light/dark cycle, 22°C), with *ad libitum* access to food and water. Animals were maintained 5–6 per cage with genotype segregated with enrichment as the form of small cylinder “cocoon,” which offer the animal the possibility to fulfill their natural nesting instinct. The animals were used in compliance with European standards for the care and use of laboratory animals and experimental protocols approved by the local Animal Ethical Committee (agreement APAFIS#2264-2015101320441671 from CEEA75, Lille, France).

### MSX-3 Treatment

MSX-3 is a water-soluble prodrug of the potent and highly selective A_2A_R antagonist MSX-2 (Sauer et al., 2000), that crosses the blood-brain barrier (Collins et al., 2010). The drug was administered through drinking water at 0.3 g/L, a dose previously shown to provide benefit in Tau transgenic mice (Laurent et al., 2016). Chronic delivery at this dose achieved, in 10-month-old C57Bl6/J mice, plasma and brain concentrations of MSX-2 of about 13 nM and 50 nM, respectively (Supplementary Figure S1), compatible with A_2A_R blockade (MSX-2 K_i_ = ca. 8 nM). Animals randomized according to their body weight, were assigned to the four following experimental groups: WT/H_2_O, WT/MSX-3, APP/PS1/H_2_O and APP/PS1/MSX-3. The MSX-3 solution was kept in bottles protected from light and changed weekly. Treatment started at 3 months of age, when amyloid pathology begins and before memory impairments in APP/PS1 mice, and continued until 9–10 months of age, when mice exhibit cortical and hippocampal amyloid pathology and memory deficits. MSX-3 consumption was assessed throughout treatment for each experimental cage. In average, mice consumed 6.6 ± 0.6 ml of the MSX-3 solution per day, corresponding to an average daily intake of 2 mg of the antagonist.

### Anxiety Assessment Using Elevated Plus Maze

The elevated plus maze was used to investigate anxiety-related behavior. The apparatus consisted of a plus-shaped maze with two closed and two open arms (30 cm long × 6.5 cm wide). Mice were placed at the center of the maze with their face in the direction of a closed arm and were allowed to explore freely for 5 min. Distance moved, velocity and time spent in arms were recorded using the Ethovision XT tracking system (Noldus).

### Spatial Memory Assessment Using the Morris Water Maze Task

Spatial memory abilities were evaluated in the standard hidden platform (PF) acquisition and retention version of the Morris Water Maze as previously described (Laurent et al., 2016). A 100-cm circular pool was filled with water, opacified with non-toxic white paint and kept at 21°C. A 10-cm round PF was hidden 1 cm beneath the surface of the water at a fixed position. Four positions around the edge of the tank were arbitrarily designated 1, 2, 3 and 4, thus dividing the tank into four quadrants (clockwise): target (hidden-PF contained), adjacent 1, opposite and adjacent 2. During the learning procedure, each mouse was given four swimming trials per day (15 min inter-trial interval) for five consecutive days. The start position (1, 2, 3, or 4) was pseudo-randomized across trials. A trial consisted of placing the mouse into the water facing the outer edge of the pool in one of the virtual quadrants and allowing it to escape to the hidden PF. A trial terminated when the animal reached the PF where it was allowed to remain for 15 s. If the animal failed to find the target before 120 s, it was manually guided to the PF where it was allowed to stay for 15 s. After completion of a trial, mice were removed from the pool and placed back to their home cages. Distance traveled to locate the hidden escape PF (path length) and swimming speed (i.e., velocity, as a measure of possible motor defects that could interfere with their ability to perform in this task) were recorded using the Ethovision XT tracking system (Noldus). Seventy-two hours following the acquisition phase, a probe trial was conducted. During this probe trial (60 s), the PF was removed and search pattern of the mice was tracked again. Proportion of time spent in the target quadrant (T) vs. averaged non-target quadrants (O) was determined.

### Sacrifice and Brain Tissue Preparation

Sacrifice of animals took place in the afternoon. Mice were deeply anesthetized with pentobarbital sodium (50 mg/kg, i.p.), then transcardially perfused with cold NaCl (0.9%). Brains were removed and one half of the hemisphere were post-fixed in 4% paraformaldehyde fixative in PBS (pH 7.4) for a week at 4°C and transferred to 30% sucrose solution overnight before being frozen. Coronal brains sections (35 μm) were obtained using a Leica cryostat. Free-floating sections were selected according the stereological rules, with the first section taken at random and every ninth sections afterwards and were stored in PBS-azide (0.2%) at 4°C. Cortex and hippocampus of the other hemisphere were dissected using a coronal acrylic slicer (Delta Microscopies) at 4°C and stored at −80°C until use.

### Immunohistochemistry and Image Analysis

Antibodies used in this study are listed in Table 1. For Aβ immunohistochemistry (IHC), sections were pretreated with 80% formic acid for 3 min and were permeabilized with 0.2% Triton X-100/sodium phosphate buffer. Sections were then blocked with 10% “Mouse On Mouse” Kit serum (Vector Laboratories) for 1 h before incubation with mouse biotinylated anti-Aβ antibody (6E10) at 4°C overnight. After washing in PBS, the sections were incubated with the ABC kit (Vector Laboratories) for 2 h and developed using DAB (Sigma). Images were acquired using Leica ICC50 HD microscope. Quantification of the 6E10 staining intensity was performed using Mercator software (Explora Nova, Mountain View, CA, USA). The number of plaques, the average plaque size and the plaque burden, expressed as percentage of analyzed area, were calculated in the cortex and hippocampus of the APP/PS1 mice. For immunofluorescence studies, coronal brain sections were washed with sodium phosphate buffer and permeabilized with 0.2% Triton X-100/sodium phosphate buffer. Sections where blocked with normal goat serum (1/100; Vector Laboratories) before incubated with an anti-GFAP antibody (Table 1) at 4°C overnight. Primary antibody was detected with Alexa Fluor 488 or 633 goat anti-rabbit IgG (1/500, Thermo Fisher). After washes, sections were blocked with donkey serum (1/100; Sigma) and were incubated for 48 h at 4°C with an anti-A_2A_R antibody followed by an incubation with Alexa Fluor 595 donkey anti-guinea pig IgG (1/500; Jackson) antibody for 2 h at RT. To visualize amyloid plaques, sections were pretreated with 80% formic acid for 3 min, blocked with 10% of Mouse On Mouse Kit serum (Vector Laboratories) for 1 h before incubated in mouse biotinylated anti-Aβ antibody at 4°C overnight. Sections were then incubated with streptavidin Alexa Fluo 488 conjugate (1/1000; Thermo Fisher Scientific). Sections were finally incubated with DAPI (1/5000; Sigma-Aldrich) for 10 min and treated for 10 min in 0.3% Suden Black (Sigma-Aldrich) with 70% ethanol to block autofluorescence. Images were acquired using a Zeiss LSM 710 confocal laser-scanning microscope to define co-localization of A_2A_R with glial markers and amyloid plaques. 3D reconstruction of 2D confocal z stacks was performed using Imaris software (Bitplane, South Windsor, CT, USA).

**Table 1:** Antibodies used in this study.

Name	Epitope	Type	Origin	Provider	Dilution
Anti-A_2A_R	CTER 33aa	Poly	Guinea Pig	Frontier Institute	1/200
Anti-GFAP	GFAP	Poly	Rabbit	Dako	1/1000
Anti-NeuN	NeuN	Mono	Mouse	Merck Mllipore	1/500 (IHC) 1/1000 (WB)
Anti-Aβ 1-16 (6E10)	Total Aβ (3-8 aa)	Mono	Mouse	Biolegend	1/1000
Anti-APP (C17)	Cter part of APP, CTFs	Poly	Rabbit	Home Made	1/5000
Anti-GluR1	Hulan GluR1 (840–850 aa)	Mono	Rabbit	Merck Mllipore	1/2000
Anti-phospho-GluRl (Ser831)	pSer831	Mono	Rabbit	Merck Mllipore	1/1000
Anti-phospho-GluR1 (Ser845)	pSer845	Mono	Rabbit	Merck Millipore	1/1000
Anti-GluR2	Mouse GluR2 (150–250 aa)	Poly	Rabbit	Abcam	1/5000
Anti-phospho-GluR2 (Ser88O)	pSer88O	Poly	Rabbit	Abcam	1/5000
Anti-NR2B	Mature NR2B (1437–1456 aa)	Poly	Rabbit	Cell Signaling	1/1000
Anti-phospho-NR2B (Tyr1472)	pTyr1472	Poly	Rabbit	Cell Signaling	1/1000
Anti-phospho-NR2B (Tyr1480)	pTyrl480	Poly	Rabbit	Thermo Fisher Scientific	1/1000
Anti-Munc-l8-1 (Muncl8)	Cter (577–594 aa)	Poly	Rabbit	Sigma	1/2000
Anti-PSD95	PSD95	Poly	Rabbit	Cell Signaling	1/1000
Anti-Spinophiline	Spinophiline	Poly	Rabbit	Merck Mllipore	1/1000
GAPDH	Hutnan GAPDH (FL 1–335)	Poly	Rabbit	Santa Cruz Biotechnology	1/1000

*Abbreviations: Mono, monoclonal; Poly, polyclonal; IHC, dilution used in Immunohistochemistry; WB, dilution used in Western blotting; GFAP, glial fibrillary acidic protein; NeuN, neuronal nuclear; APP, amyloid precursor protein; GAPDH, glyceraldehyde-3-phosphate*.

### Western Blots

For all biochemical experiments, tissue was homogenized in 200 μL Tris buffer (pH 7.4) containing 10% sucrose and protease inhibitors (Complete; Roche Diagnostics GmbH), sonicated, and kept at −80°C until use. Protein amounts were evaluated using the BCA assay (Pierce). Protein amounts were evaluated using the BCA assay (Pierce), subsequently diluted with LDS 2X supplemented with reducing agents (Invitrogen) and then separated on 4%–12% NuPage Novex gels (Invitrogen). Proteins were transferred to nitrocellulose membranes, saturated (5% non-fat dry milk or 5% BSA) in TNT (Tris 15 mM pH 8, NaCl 140 mM, 0.05% Tween) and incubated with primary (see Table 1) overnight and then corresponding secondary antibodies (peroxidase labeled horse anti-rabbit 1/5000 or anti-mouse 1/50,000, Vector Laboratories). Immunoreactivity was visualized using chemiluminescence kits (ECL^TM^, Amersham Bioscience) and a LAS3000 imaging system (Fujifilm). Results were normalized to GAPDH and quantifications were performed using ImageJ software (Scion Software).

### mRNA Extraction and Quantitative Real-Time RT-PCR Analysis

Total RNA was extracted from hippocampi and cortex, and purified using the RNeasy Lipid Tissue Mini Kit (Qiagen, France). One microgram of total RNA was reverse-transcribed using the Applied Biosystems High-Capacity cDNA reverse transcription kit. Quantitative real-time RT-PCR analysis was performed on an Applied Biosystems Prism 7900 System using Power SYBR Green PCR Master Mix. The thermal cycler conditions were as follows: hold for 10 min at 95°C, followed by 45 cycles of a two-step PCR consisting of a 95°C step for 15 s followed by a 60°C step for 25 s. Sequences of primers used are given in Table 2. Cyclophilin A was used as internal control. Amplifications were carried out in triplicate and the relative expression of target genes was determined by the ΔΔCT method.

**Table 2:** Primer sequences used in this study.

Name	Access number	Primer FW	Primer R	Amplicon size
GFAP	NM_001131020.1	cgcgaacaggaagagcgcca	gtggcgggccatctcctcct	104
Cd68	NM_009853.1	gacctacatcagagcccgagt	cgccatgaatgtccactg	95
TLR2	NM_011905.3	ggggcttcacttctctgctt	agcatcctctgcgatttgacg	110
CCL3	NM_011337.2	tgcccttgctgttcttctct	gtggaatcttccggctgtag	112
CCL5	NM_013653.3	ctcactgcagccgccctctg	ccgagccatatggtgaggcagg	51
Cyclophilin	NM_008907.1	agcatacaggtcctggcatc	ttcaccttcccaaagaccac	126

*Abbreviations: FW, Forward; R, Reverse*.

### ELISA Measurements

Brain levels of human Aβ1-40 and Aβ1-42 were measured using ELISA kits (Invitrogen, Carlsbad, CA, USA; IBL-International, Hamburg, Germany) following manufactured’ instructions. Briefly, for hippocampal and cortical samples, 20 μg of protein were diluted in Guanidine/Tris buffer (Guanidine HCl 5 M and Tris 50 mM pH 8), sonicated and incubated for 1 h at 4°C under agitation. Samples were then diluted in a BSAT-DPBS solution (KCl, KH_2_PO_4_, NaCl, Na_2_HPO_4_, BSA 5%, Tween-20 0.03% pH 7.4). The homogenates were centrifuged at 12,000 *g* for 15 min at 4°C. Supernatants were collected for the analysis of Aβ1-40 and Aβ1-42 by colorimetric immunoassays. Absorbance was measured by Multiskan Ascent counter (ThermoLab Systems). The normalized amounts of Aβ were expressed as pg/mL.

### Evaluation of Microglial Phagocytosis

The effect of acute A_2A_R blockade on microglial phagocytosis was quantified in an *in situ* live cerebral slices assay similar to what has been described (Krabbe et al., 2013; Savage et al., 2015). APP/PS1 mice were crossbred with Csfr1r-EGFP mice (Sasmono et al., 2003) to readily visualize microglia. Coronal acute cerebral slices with a thickness of 130 μm were prepared from 12 month-old APP/PS1-Csf1r-EGFP mice and Csf1r-EGFP wildtype littermates using a vibratome (Leica VT1200 S). Acute slices were pre-incubated with the indicated concentration of MSX-3 (10–5000 nM) or vehicle for 60 min in artificial cerebrospinal fluid (aCSF) under constant carbogen saturation (three acute cerebral slices per condition). Live acute slices were then incubated with FCS-coated fluorescent microspheres (2 μm diameter, flash red, Bang Laboratories Inc.) at a concentration of 1.1 × 10^7^ microspheres/mL for 60 min at 37°C in HBSS together with the indicated concentration of MSX-3 or vehicle. Slices were washed and fixed in 4% PFA. Aβ dense-core plaques were stained with 0.001% thiazine red (Sigma Aldrich) in PBS. Five corresponding regions of interest (ROIs) were recorded in the isocortex of each cerebral slice using a Nikon eclipse Ti-E confocal microscope (60× objective, 20 μm z stack, 1 μm z slice interval) and the phagocytic index, i.e., quotient between number of fluorescent microspheres internalized by GFP-positive plaque-associated microglia and the total number of plaque-associated microglia per ROI was quantified by an investigator blinded to the treatment condition.

### Transcriptomic Analysis (Agilent Microarray)

Total RNA yield and quality were assessed on the Agilent 2100 bioanalyzer (Agilent Technologies, Massy, France). One color whole Mouse (074809_D_F_20150624 slides) 60-mer oligonucleotides 8× 60k microarrays (Agilent Technologies) were used to analyze gene expression. Six biological replicates for each condition were prepared for a total of 48 samples (Cortex or Hippocampus of WT-H_2_O/WT-MSX-3/APP- H_2_O/APP-MSX-3). cRNA labeling, hybridization and detection were carried out according to supplier’s instructions (Agilent Technologies). For each microarray, Cyanine 3-labeled cRNA were synthesized with the low input QuickAmp labeling kit from 50 ng of total RNA. RNA Spike-In were added to all tubes and used as positive controls of labeling and amplification steps. The labeled cRNA were purified and 600 ng of each cRNA were then hybridized and washed following manufacturer’s instructions. Microarrays were scanned on an Agilent G2505C scanner and data extracted using Agilent Feature Extraction Software^©^ (FE version 10.7.3.1). Microarray data are available through the GEO depository from NCBI (accession no. GSE113141).

The statistical analyses were performed with Genespring^®^ software version GX13.0 (Agilent Technologies). Microarrays have been normalized to the 75th percentile. Probes below background under all conditions were removed from the analysis. Differentially expressed genes were identified by a moderated *t*-test with correction of multiple tests by the Benjamini Hochberg (BH) method. We selected significantly deregulated probes with a corrected *p*-value of less than 0.05 and with an expression differential of at least 1.5× (FC1.5). Differentially expressed probes were further analyzed in term of molecular function and biological process using the Ingenuity Pathways Analysis (Qiagen Inc.) software.

### Statistics

Results are expressed as means ± SEM. Differences between mean values were determined using the Student’s *t*-test, Two Way-analysis of variance (ANOVA) or One Way-ANOVA followed by a *post hoc* Fisher’s LSD test using Graphpad Prism Software. *P* values < 0.05 were considered significant.

## Results

### A_2A_R Is Overexpressed by Astrocytes in APP/PS1 Transgenic Mice

**Figure 1:**
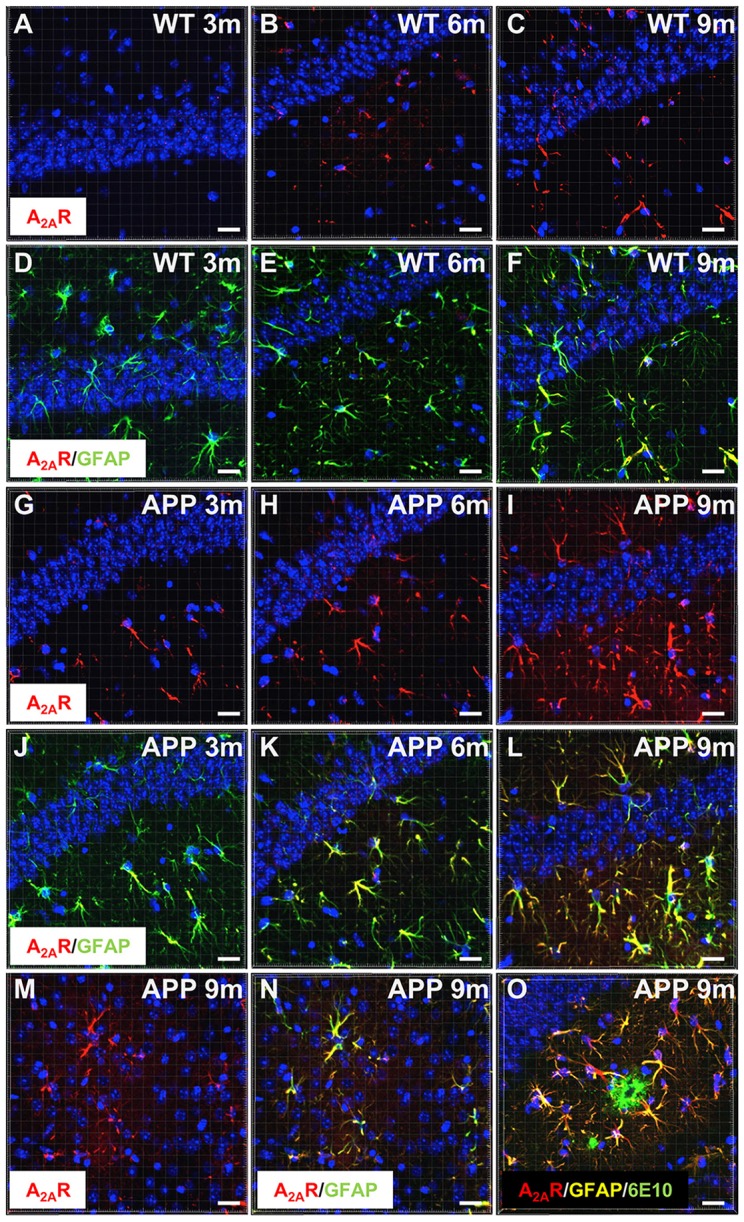
Astrocytic upsurge of A_2A_Rs in APP/PS1 mice. Representative photomicrographs of hippocampal immunostaining for the A_2A_ receptor (A_2A_R; red) **(A–C,G–I)** and merged with the astrocyte marker GFAP (green) **(D–F,J–L)** from WT mice **(A–F)** and the APP/PS1 mice **(G–L)**, at different ages: 3 months **(A,D,G,J)**, 6 months **(B,E,H,K)**, and 9–10 months **(C,F,I,L)**. Representative photomicrographs of A_2A_R expression (red) **(M)** and the merged with GFAP marker (green; **N**) in the cortex of 9-month-old APP/PS1 mice. Representative photomicrograph of A_2A_R expression (red) GFAP (yellow) and 6E10-positive amyloid plaque marker (green) in the hippocampus of 9 month-old APP/PS1 mice **(O)**. Cell nuclei were labeled with DAPI (blue). Scale bar = 20 μm.

We first examined A_2A_R expression levels in APP/PS1 mice using IHC and confocal microscopy. Similar to what has been previously described in AD patients and in different APP models (Orr et al., 2015, 2018; Lee et al., 2018), we observed that APP/PS1 mice exhibit a progressive hippocampal upsurge of A_2A_R immunoreactivity (Figures 1G–I), as compared to WT littermates (Figures 1A–C). Importantly, A_2A_R immunopositive signal observed in the striatum and hippocampus of APP/PS1 mice was found abolished in APP/PS1 animals with genetically deleted A_2A_R (Supplementary Figure S2) supporting the specificity of the A_2A_R immunostaining provided in Figure 1. In APP/PS1 mice, increased A_2A_R expression was observed in astrocytes in the hippocampus, starting at 3 months of age, and the cortex, starting at 9 months of age (Figures 1D–F,J–N). Astrocytic A_2A_R overexpression was not found exclusively in the vicinity of 6E10-positive plaques but also in reactive astrocytes located at larger distance to plaques (Figure 1O). These data therefore indicate that the development of amyloid pathology elicits astrocytic A_2A_R upsurge in APP/PS1 mice.

### A_2A_R Pharmacological Blockade Prevents Spatial Memory Impairments in APP/PS1 Transgenic Mice

**Figure 2:**
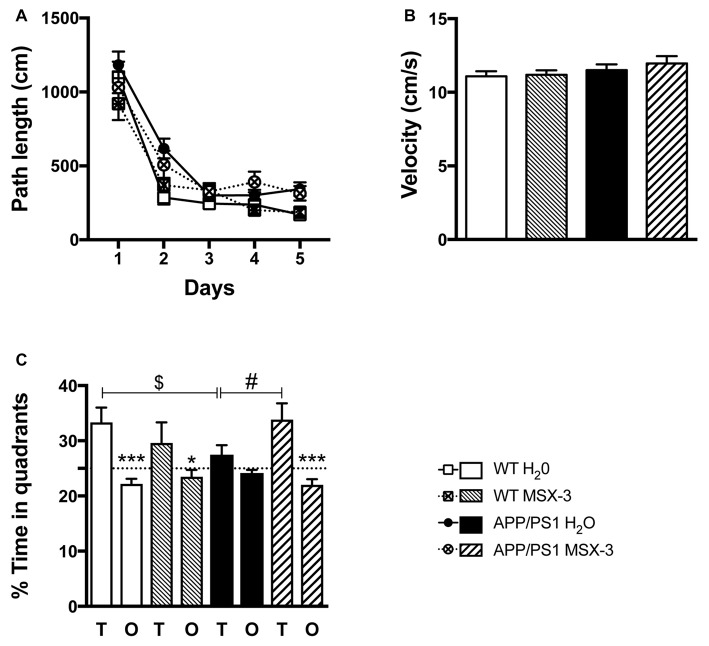
A_2A_R blockade prevents spatial memory impairments in APP/PS1 transgenic mice. Effect of MSX-3 treatment on spatial learning and memory using the Morris water-maze task. **(A)** Learning, as indicated by the equivalent path length needed to find the hidden platform (PF). At day 2, APP/PS1 mice exhibited a slight but significant higher path length as compared to WT animals (***p < 0.001 vs. WT H_2_O using One-way analysis of variance (ANOVA) followed by LSD Fisher *post hoc* test). **(B)** All genotypes exhibited a comparable velocity in the maze, suggesting no motor deficits. **(C)** Spatial memory was assessed 72 h after the last day of learning. Results represent the percentage of time spent in the target (T) vs. non-target (O) quadrants. WT mice (both treated with water or MSX-3) spent significantly more time in the T quadrant, indicative of a preserved spatial memory. While APP/PS1 mice exhibited spatial memory deficits as underlined by their lack of preference for the T quadrant, APP/PS1-MSX-3 treated mice behaved as WT mice, suggesting a rescue of memory impairment. *p < 0.05 ***p < 0.001 T vs. O; ^$^*p* < 0.05 WT vs. APP/PS1; ^#^*p* < 0.05 APP/PS1-H_2_O vs. APP/PS1 MSX-3 using One-way ANOVA followed by LSD Fisher *post hoc* test; *N* = 12–16 per group; Results are expressed as mean ± SEM.

In the present experimental paradigm, APP/PS1 mice and littermate controls were treated with MSX-3 in drinking water at 0.3 g/L, using a treatment paradigm we previously described to promote a significant benefit in a model of AD-like Tau pathology (Laurent et al., 2016). Mice were treated from 3 to 9-10 months of age, i.e along the development of amyloid pathology and cognitive deficits in this transgenic strain. At completion of the treatment, we evaluated the impact of the chronic A_2A_R blockade on anxiety and spatial learning and memory using, respectively, the Elevated Plus Maze and the Morris Water Maze. We analyzed the impact of MSX-3 upon anxiety using the Elevated Plus Maze task. As shown in Supplementary Figure S3, we did not find any significant impact of MSX-3 upon velocity, distance moved and percentage of time spent in both closed and open arms (*p* > 0.05), suggesting that the treatment did not significantly impact anxiety behavior in both WT and APP/PS1 mice. Regarding Morris Water Maze, during the training phase, all groups showed a decrease in path length across trials (*p* < 0.05, two-way ANOVA). APP/PS1 animals demonstrated a slight learning deficit at day 2 (*p* = 0.0002, Two-Way ANOVA followed by Fisher LSD *post hoc* test) with no impact of the treatment (*p* > 0.05; Figure 2A). Neither APP/PS1 genotype nor the MSX-3 treatment influenced mouse velocity (*p* > 0.05; Figure 2B). Seventy-two hours following acquisition, a probe trial was performed to evaluate spatial memory. Regardless of treatment (H_2_O or MSX-3), WT littermates exhibited a significant preference for the target quadrant (Figure 2C; WT H_2_O: *p* = 0.0003; WT MSX3: *p* = 0.039 vs. O quadrants using One-way ANOVA followed by LSD Fisher *post hoc* test). As expected at this age, APP/PS1 H_2_O mice showed spatial memory deficits, evidenced by the absence of preference for the target vs. the non-target (O) quadrants (Figure 2C; *p* > 0.05 vs. O quadrants using One-way ANOVA followed by LSD Fisher *post hoc* test). ANOVA analysis also indicated that the percentage of time spent in target quadrant for APP/PS1 H_2_O mice was significantly reduced as compared to WT H_2_O animals (*p* = 0.037). In sharp contrast, the blockade of A_2A_R by MSX-3 significantly alleviated spatial memory impairment in APP/PS1 mice, as demonstrated by a significant preference of MSX-3-treated APP/PS1 mice for the target quadrant (Figure 2C, *p* = 0.0002 vs. O quadrants using One-way ANOVA followed by LSD Fisher *post hoc* test). ANOVA analysis also indicated that APP/PS1 mice treated with MSX-3 spent a higher percentage of time in the target quadrant as compared to water-treated APP/PS1 animals (*p* = 0.027; Figure 2C). Altogether, these data indicated that early onset chronic A_2A_R blockade prevents the development of spatial memory alterations in APP/PS1 mice.

### Effect of Chronic A_2A_R Blockade on Hippocampal Synaptic Markers in APP/PS1 Transgenic Mice

To evaluate whether the beneficial effects of A_2A_R blockade on spatial memory in the APP/PS1 mice could be ascribed to changes in the expression of hippocampal synaptic markers, we performed western blot evaluation of neuronal, pre/post-synaptic proteins as well as glutamatergic receptors. While MSX-3 did not modulate the expression of the studied neuronal and postsynaptic markers (NeuN, spinophiline and PSD95; *p* > 0.05, vs. APP/PS1 H_2_O mice using Student’s *t*-test; Supplementary Figure S4A) nor the expression and phosphorylation of AMPA and NMDA receptor subunits (*p* > 0.05, vs. APP/PS1 H_2_O mice using Student’s *t-test*; Supplementary Figure S4B), we found that MSX-3 treatment significantly increased the hippocampal expression of the presynaptic marker Munc-18 in the APP/PS1 mice treated with MSX-3 (*p* = 0.037 vs. APP/PS1 H_2_O using Student’s *t-test*; Supplementary Figure S4A). Notably, none of the markers studied was found modified in the cortex of APP/PS1 MSX-3 animals (not shown).

### Effect of Chronic A_2A_R Blockade on Cortical and Hippocampal Transcriptome of APP/PS1 Transgenic Mice

In order to provide potential molecular insights on how chronic A_2A_R blockade by MSX-3 improves memory of APP/PS1 mice, we evaluated gene expression changes in the cortex and hippocampus in the different groups of mice using Agilent technology. First, we determined gene expression changes between APP/PS1 H_2_O vs. WT H_2_O. In the cortex, 519 probes were significantly changed (BH adjusted *p*-values < 0.05) with a major effect (FC > 10) for Clec7a, Itgax, Cst, Ccl3, Ccl4 and Cxcl10 (Supplementary Table S1). In the hippocampus, 125 probes were significantly altered (BH adjusted *p*-values < 0.05; Supplementary Table S3) and we found the same highly modified genes, indicating highly concordant results between structures. The study of molecular functions by Ingenuity Pathway Analysis highlighted a significant number of deregulated genes in pathways related to immune functions (Supplementary Tables S2, S4), in accordance with the known link between amyloid load and the development of parenchymal neuroinflammation (Heneka et al., 2015 for review), which is known to favor cognitive deficits (Marciniak et al., 2015; Laurent et al., 2018 and references herein). Using quantitative PCR analysis, we validated several neuroinflammatory markers (GFAP, CD68, TLR2, CCL5 and CCL3) in both the cerebral cortex and the hippocampus of APP/PS1 H_2_O mice as compared to WT H_2_O animals (*p* < 0.001 vs. WT using One Way ANOVA followed by LSD Fisher *post hoc* text; Supplementary Figures S5A,B). We were then interested in comparing gene expression changes in the mouse groups treated with MSX-3. Notably, A_2A_Rs were shown to modulate the function and activation of brain innate immune cells (i.e., microglia and astrocytes; Cunha, 2016 for review) and blockade of these receptors may resolve brain neuroinflammation (Rebola et al., 2011; Laurent et al., 2016). Surprisingly, no significant effect of MSX-3, particularly for markers associated to immune functions (see also Supplementary Figures S5A,B), was observed in the cortex or hippocampus of WT or APP/PS1. Altogether, these data suggest that memory improvement in APP/PS1 treated with MSX-3 is associated with a weak transcriptional effect and a notable absence of impact on neuroinflammation.

### Effect of Chronic A_2A_R Blockade on Amyloid Burden in APP/PS1 Transgenic Mice

**Figure 3:**
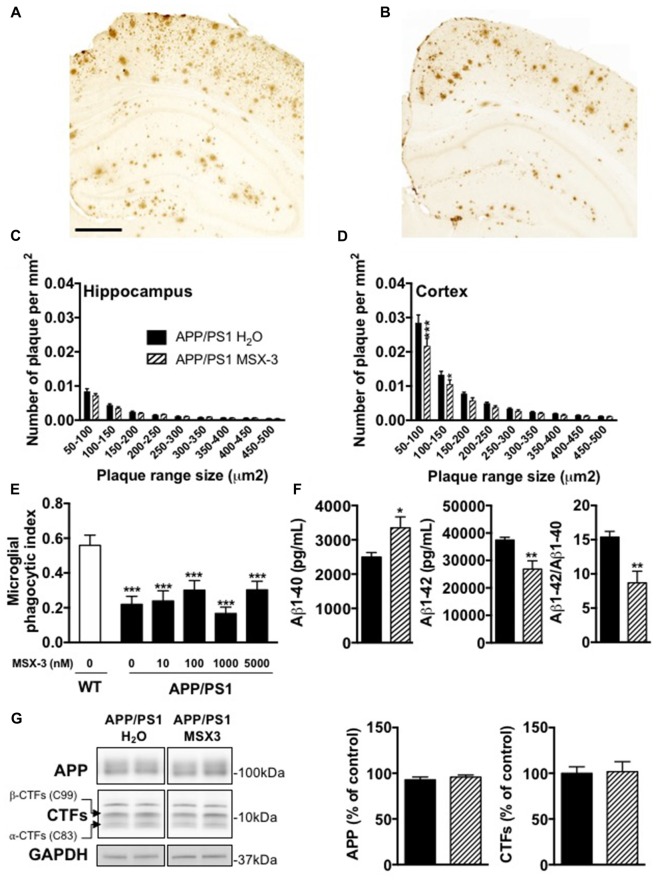
Impact of MSX-3 treatment on amyloid load, Aβ levels and phagocytic capacity of microglia in APP/PS1 mice. Representative images of 6E10 staining in the brains of 10 months old APP/PS1 treated with water **(A)** or MSX-3 **(B)**. Scale bar = 500 μm. Distribution of amyloid plaques size were examined in the hippocampus **(C)** and cortex **(D)** of APP/PS1 mice. We found that the treatment with MSX-3 significantly reduced, in the cortex **(D)** but not in the hippocampus **(C)**, the density of plaques of lower size (between 50–150 μm^2^) as compared with APP/PS1 H_2_O animals (50–150 μm^2^, *p* < 0.001; 100–150 μm^2^, *p* < 0.05 vs. APP/PS1 H_2_O using Two-Way ANOVA followed by LSD Fisher *post hoc* test; *N* = 7–9/group). **(E)** Effect of acute MSX-3 treatment on microglial phagocytic index determined in an *in situ* live cerebral slice assay of 12-month-old APP/PS1-Csf1r-EGFP mice and Csf1r-EGFP wildtype littermates after 60 min pre-incubation with the indicated concentration of MSX-3. Datasets were tested for significance with the One-way ANOVA and represent data from three independent experiments. ***p < 0.001 vs. WT **(F)** As measured by ELISA, MSX-3 treatment decreased Aβ1–42 levels in the cortex of APP/PS1 mice while Aβ1–40 levels was found increased. Overall, the Aβ1–42/Aβ1–40 ratio was found significantly reduced by the A_2A_ antagonist treatment (*p < 0.05, **p < 0.01 vs. APP/PS1 H_2_O using Student’s *t*-test; *N* = 7–11/group). **(G)** Western blot analysis performed in cortex of water and MSX-3 treated APP/PS1dE9 mice did not revealed any change in APP and Carboxyterminal fragments (CTFs) expression (*N* = 6/group). Results are expressed as mean ± SEM.

Age-dependent spatial memory impairment in APP/PS1 has been shown to correlate with increased brain amyloid burden (Savonenko et al., 2005; Garcia-Alloza et al., 2006; Zhang et al., 2011). Previous studies demonstrated that long-term caffeine treatment mitigates memory defects in APPsw mice, while reducing brain Aβ production (Arendash et al., 2006, 2009). While this is still controversial (Lu et al., 2016), other data indicated that A_2A_R might impact on the production of Aβ *in vitro* (Nagpure and Bian, 2014). Altogether, these data supported a possible involvement of A_2A_Rs in Aβ production and/or accumulation and, therefore upon memory. Using 6E10 IHC, we first performed the analysis of cortical and hippocampal Aβ plaque load in APP/PS1 mice treated with water or MSX-3 (Figures 3A,B). We found that the treatment with MSX-3 significantly reduced, in the cortex (Figure 3D) but not in the hippocampus (Figure 3C), the density of plaques of lower size (between 50–150 μm^2^) as compared with APP/PS1 H_2_O animals (50–150 μm^2^, *p* < 0.001; 100–150 μm^2^, *p* < 0.05 vs. APP/PS1 H_2_O using Two-Way ANOVA followed by LSD Fisher *post hoc* test). Microglia surrounding amyloid plaques have an impaired phagocytic capacity (Krabbe et al., 2013; Savage et al., 2015). Since adenosine signaling and A_2A_R activation has been shown to impair microglial phagocytosis in cell culture and *in situ* (Orr et al., 2009; Bulavina et al., 2013), we tested the possibility that MSX-3 could enhance the phagocytic capacity of microglia, thereby potentially explaining the moderate plaque reduction seen in the cortex of chronically MSX-3-treated APP/PS1 animals. To test whether A_2A_R blockade has an immediate effect on microglial phagocytosis as has been shown for other microglial functions (Gyoneva et al., 2014, 2016), we used an *in situ* live acute slice assay and evaluated whether acute A_2A_R blockade with MSX-3 in live cerebral slices could enhance the phagocytic capacity of microglia surrounding amyloid plaques. However, although our results confirmed that 12-month-old APP/PS1 mice exhibit a reduced phagocytic activity of microglia as compared with littermate controls, acute treatment of acute slices from APP/PS1 mice with MSX-3 of up to 5 μM failed to normalize phagocytosis of plaque-associated microglia (Figure 3E), suggesting that acute A_2A_R blockade does not significantly affect microglial phagocytosis.

Next, we measured the concentration of Aβ1-42 and Aβ1-40 in the cortex and the hippocampus. The cortex of MSX-3-treated APP/PS1 mice exhibited a significant decrease of Aβ1-42 levels (*p* = 0.0011 vs. APP/PS1 H_2_O using Student’s *t*-test; Figure 3F) while Aβ1-40 levels were increased (*p* = 0.012 vs. APP/PS1 H_2_O using Student’s *t*-test; Figure 3F). Overall, in the cortex, the Aβ42/Aβ40 ratio was found to be significantly reduced in APP/PS1 MSX-3 mice as compared to APP/PS1 H_2_O animals (*p* = 0.0015 vs. APP/PS1 H_2_O using Student’s *t*-test; Figure 3F). These cortical changes were not accompanied by a modification of APP expression or C-terminal fragments (CTF) of APP (Figure 3G). Also, none of the parameters studied was found changed in the hippocampus of APP/PS1 MSX-3 animals (*p* > 0.05, Student’s *t*-test; not shown). Altogether, these data indicated that early and chronic A_2A_R blockade reduces the development of cortical amyloid burden in APP/PS1 mice.

## Discussion

The present study demonstrates that an early, chronic and long-term treatment with a specific A_2A_R antagonist, starting from an asymptomatic stage, prevents spatial memory impairments and reduces, at least in part, the development of amyloidogenesis in APPswe/PS1dE9 mice. These data extend previous findings showing that short-term treatment with different antagonists (KW6002 p.o. and SCH58261 i.p.; Viana da Silva et al., 2016; Orr et al., 2018; Silva et al., 2018) improves memory of three different models of amyloidogenesis (APP/PS1 and hAPP-J20). Here, we also provide the first experimental evidence that, *in vivo*, early blockade of A_2A_Rs can reduce brain amyloid levels. Taken together with previous studies demonstrating the ability of A_2A_R blockade to reduce Tau hyperphosphorylation and associated cognitive decline (Laurent et al., 2016; Zhao et al., 2017), these data support that A_2A_R signaling is a target of interest in AD.

We found a progressive age-dependent astrocytic A_2A_R upsurge in the hippocampus of APP/PS1dE9 mice, in accordance with recent studies demonstrating similar receptor dysregulation in several mouse models of cerebral amyloidosis (hAPP-J20, APP KI; Orr et al., 2015, 2018; Lee et al., 2018). A_2A_R dysregulation likely contributes to memory deficits since conditional astrocytic-specific A_2A_R deletion (Orr et al., 2015) or pharmacological blockade (Orr et al., 2018; the present data) of the receptors improve memory performance in APP mice. The idea that astrocytic upregulation of A_2A_R might be detrimental for memory in AD models is in line with its physiological ability to control both glutamate and GABA uptake by these glial cells (Nishizaki et al., 2002; Matos et al., 2012; Cristóvão-Ferreira et al., 2013). In support of this, we recently demonstrated that A_2A_R deletion improves memory while normalizing glutamate/GABA balance in the hippocampus of Tau transgenic mice (Laurent et al., 2016). Interestingly, recent data demonstrated that specific deletion of astrocytic A_2A_Rs leads to neuronal adaptative changes in glutamatergic synapses, characterized by increased evoked release of glutamate from nerve terminals or enhanced density of NR2B (Matos et al., 2015). Further, it remains also possible that modulation of astrocytic A_2A_Rs impacts the function of synaptic A_2A_Rs. Indeed, deletion of A_2A_R in astrocytes has been shown to enhance the ability of A_2A_R agonist CGS2160 to promote glutamate release by synaptosomes (Matos et al., 2015). Therefore, astrocytic A_2A_R upsurge is likely prone to favor synaptic dysfunctions leading to memory deficits in APP/PS1 mice. Further, we cannot rule out that the benefit afforded by the pharmacological A_2A_R blockade in APP/PS1 mice also result from the specific blockade of A_2A_R on other receptor subpopulations, notably at the neuronal level. Indeed, neuronal upsurge of A_2A_R have been observed in the brain of AD patients (Temido-Ferreira et al., 2018) and recent work demonstrated that APP/PS1 mice exhibit a significant increase of A_2A_R binding on synaptic hippocampal membranes (Viana da Silva et al., 2016; Silva et al., 2018). It is thus highly conceivable that APP/PS1 mice exhibit both astrocytic and synaptic A_2A_R upsurge, the latter being probably difficult to capture using classical immunohistofluorescence. In this regard, neuronal/synaptic modulation of A_2A_Rs could also play an instrumental role in synaptic and memory deficits of APP mice. Indeed, mimicking neuronal A_2A_R upsurge using conditional transgenic models or optogenetically enhancing intracellular signaling of the receptor was sufficient to promote plasticity and memory deficits (Giménez-Llort et al., 2007; Li et al., 2015; Batalha et al., 2016). Thus, it is likely that the pharmacological blockade of A_2A_Rs also acts at the neuronal level to normalize memory deficits. It is also important to emphasize that the link between synaptic A_2A_R upregulation and memory deficits goes far beyond the AD context, as demonstrated by the laboratories of Rodrigo Cunha and Luisa Lopes. For instance, synaptic A_2A_R have been found dysregulated in models of aging, stress or depression with behavioral manifestations normalized by A_2A_R receptor antagonists (Lopes et al., 1999; Rebola et al., 2003; Batalha et al., 2013; Kaster et al., 2015; Machado et al., 2017). Interestingly, in regard to presumable neuronal-based mechanisms, we observed, among the synaptic markers studied, an enhanced level of Munc-18 protein as seen by Western blot. Munc-18 is a neuronal (presynaptic) protein required for synaptic vesicles exocytosis (Carr and Rizo, 2010) and is considered as an important regulator of synaptic transmission and presynaptic strength (Toonen and Verhage, 2007; Genc et al., 2014), which is a crucial process in neuronal information processing and memory formation (Abbott and Regehr, 2004). Thus, enhancement of hippocampal Munc18 expression could also therefore contribute to memory improvement promoted by MSX-3. Besides memory impairments, it is important to mention that AD is also associated with neuropsychiatric symptoms. Recently, a study emphasized that long-term oral treatment with caffeine, a non-selective antagonist of A_2A_Rs, exacerbate some behavioral symptoms in the triple Tg AD model (Baeta-Corral et al., 2018). While we only addressed the impact of MSX-3 on anxiety in our study behavior, which appears not affected by the antagonist, it will be important, in the future and from a therapeutic perspective, to evaluate more closely the impact of long-term A_2A_R blockade on AD-related behavioral symptoms.

Our microarray data indicate that both cortex and hippocampus of 9–10-month-old APP/PS1 mice exhibit gene deregulation as compared to littermate controls. As expected, the number of deregulated genes was higher in the cortex, which harbors a more advanced pathology compared to the hippocampus in this mouse model (Kim et al., 2012). Several pathways were strongly dysregulated by the development of amyloid pathology (Suh and Checler, 2002), among them processes related to immune functions and neuroinflammation, which we confirmed by qPCR analysis. Interestingly and surprisingly, none of these pathways were impacted by MSX-3 treatment. Furthermore, chronic MSX-3 treatment of WT mice did not have a significant impact on the transcriptome either. These data indicate that, overall, chronic A_2A_R inhibition is associated with very weak transcriptional effects. Actually, to the best of our knowledge, only one study reported an evaluation of transcriptomic changes following A_2A_R blockade. This study reported that constitutive deletion of A_2A_R in the striatum of knock-out mice leads to a significant deregulation of 152 genes compared to WT littermates (Yu et al., 2009). These data are difficult to compare to our experiments since the striatum is highly enriched in A_2A_R as compared to cortex and hippocampus, with a major post-synaptic localization (Blum et al., 2003a,b; Cunha, 2016). The observation that MSX-3 improves memory of APP/PS1 mice is however in accordance with its acknowledged ability to fine tune synaptic plasticity in the hippocampus (Cunha, 2016). Recent data notably emphasized that A_2A_R blockade can lead within minutes to an improvement of plasticity deficits in APP/PS1 mice (Viana da Silva et al., 2016).

However, improved memory in APP/SP1 MSX-3 mice in our data was found to be associated with reduced amyloid load. In APP/PS1dE9 mice, amyloid burden, and particularly hippocampal and cortical Aβ42 levels, strongly correlate with spatial memory deficits (Puoliväli et al., 2002; Garcia-Alloza et al., 2006; Sipos et al., 2007; Zhang et al., 2011). Our results demonstrate that MSX-3 treatment promotes a moderate but significant reduction of amyloid plaques in the cortex of APP/PS1 mice. Importantly, a significant reduction of the Aβ1-42/Aβ1-40 ratio was also observed in the cortex of APP/PS1 mice following A_2A_R blockade. To our knowledge, this is the first report demonstrating an effect of an A_2A_R ligand on amyloid pathology *in vivo*, in agreement with previous studies showing that chronic caffeine treatment reduces brain soluble Aβ in APPsw mice (Arendash et al., 2006, 2009). The decrease of amyloid burden following A_2A_R blockade found in the cortex of APP/PS1 mice, a brain region involved in spatial navigation, could therefore explain, at least in part, the beneficial effect of MSX-3 on spatial memory. Reasons explaining a specific effect of MSX-3 on cortical amyloid pathology vs. hippocampus remain unclear. Similarly, the mechanisms underlying the changes of Aβ1-42/Aβ1-40 ratio and 6E10 immunoreactivity warrant further evaluations. However, the reduction of the Aβ1-42/Aβ1-40 is consistent with the reduction of amyloid burden, especially of small amyloid plaques. In fact, *in vitro* data indicated that Aβ42 facilitates amyloid nucleation while Aβ40 allows for Aβ elongation (Snyder et al., 1994). In the present work, small plaques, reflecting amyloid nucleation, are decreased presumably due to the reduced amount of Aβ42 peptides. It cannot also be excluded that MSX-2 might bind to Aβ and act as a direct amyloid aggregation inhibitor, which warrants further exploration in the future. Interestingly, caffeine was shown, *in vivo* and *in vitro* to reduce both Aβ1-42 and Aβ1-40 levels (Arendash et al., 2006). This contrast with our data which indicate that MSX-3 modulates Aβ1-42/Aβ1-40 ratio, reducing Aβ1-42 and enhancing Aβ1-40 levels. Aβ length is under the control of the carboxypeptidase activity of the gamma-secretase which cleaves the Aβ peptide from its carboxy-terminal region. Thus, lack of PS1 exon 9 and exon 10 generates longer Aβ peptides (Le Guennec et al., 2017). Although speculative, MSX3 could promote the carboxypeptidase activity of the gamma-secretase and thus modify the ratio of Aβ towards the production of shorter species of Aβ peptides. In line with this hypothesis, A_2A_R and gamma-secretase have been co-localized to endosomes and A_2A_Rs have been suggested to modulate the gamma-secretase activity (Lu et al., 2016). Although controversial, our results also support a regulatory activity of A_2A_R towards the gamma-secretase in APP/PS1 mouse. However, the underlying mechanism remains to be elucidated.

In conclusion, we have shown for the first time that a chronic and long-lasting treatment with an A_2A_R antagonist reduces amyloid pathology and improves memory in a model of AD. Considering previous converging studies in different models of AD (Orr et al., 2015, 2018; Laurent et al., 2016; Viana da Silva et al., 2016; Silva et al., 2018), the present findings further highlight A_2A_R as a promising therapeutic target in AD.

## Author Contributions

EF, JC, KZ, EM, YB, MS, LC, KC, SS, SE and RC performed experiments, analyzed data and corrected the manuscript. MF, YC, MH, NS, CM, AH, LB, LL and DB supervised the work, analyzed data and wrote the manuscript.

## Conflict of Interest Statement

The authors declare that the research was conducted in the absence of any commercial or financial relationships that could be construed as a potential conflict of interest.
