# Supplementary material for: Beneficial Effect of a Selective Adenosine A2A Receptor Antagonist in the APPswe/PS1dE9 Mouse Model of Alzheimer’s Disease
Source: Front Mol Neurosci. 2018 Jul 12;11:235. doi: 10.3389/fnmol.2018.00235 (PMC6052540; doi:10.3389/fnmol.2018.00235)
Supplement: Supplementary file 5 [file Image_1.PDF]

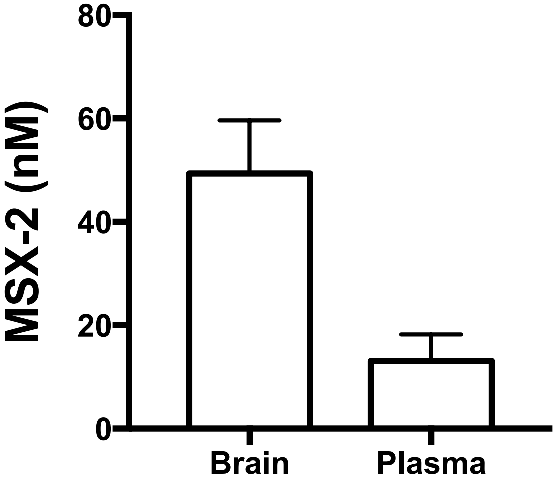

Supplementary Figure 1. Determination of MSX-2 levels in mouse plasma and brain samples following chronic treatment with MSX-3 in the drinking water. Quantitative determination of MXS-2 was performed by LCMS in plasma and brain samples of 10 months old C57Bl6/J mouse after chronic treatment of MSX-3, started at 3 months of age. Brain samples of 2-3 mice were weighed, pooled and freeze-dried. Then they were minced and subsequently extracted for 4h in 80 mL of dichloromethane using a Soxhlet extractor. The solvent was reduced to ca. 4 mL, filtered through 0.45 µm filters, and the filtrate was reduced to dryness. The residue was dissolved in 200 µL acetonitrile and subjected to LCMS measurement. Plasma samples were taken up in acetonitrile (1:10) and the mixture was homogenized in an ultrasound bath in the dark for 5 min and subsequently centrifuged at 14,000g. The supernatant was dried for 1h at 40°C in a Speedvac® (ThermoFischer Scientific). Then acetonitrile was added (100 µL) and the solution was measured by LCMS. For quantitative LCMS measurements and HPLC Dionex Ultimate 3000 (Thermo Scientific) with integrated variable wavelength was used coupled with a mass spectrometer micrOTOF-Q (Bruker) with an ESI source. An EC50/2 Nucleodur C18 Gravity 3 µM column (Macherey & Nagel) was used for HPLC separations. An HPLC gradient was run starting with 10% acetonitrile and 90% water containing 2 mM ammonium acetate. The gradient started after 1 min reaching 100% acetonitrile after 9 min. The column was flushed with 100% acetonitrile for another 5 min. Then 10 µL of sample solution was injected at a flow rate of 0.3 mL/min. Positive full scan MS was observed from 50 to 1000 m/z. For identification and quantification of MSX-2 the extract ion chromatogram (EIC) of 395.17 m/z was used.

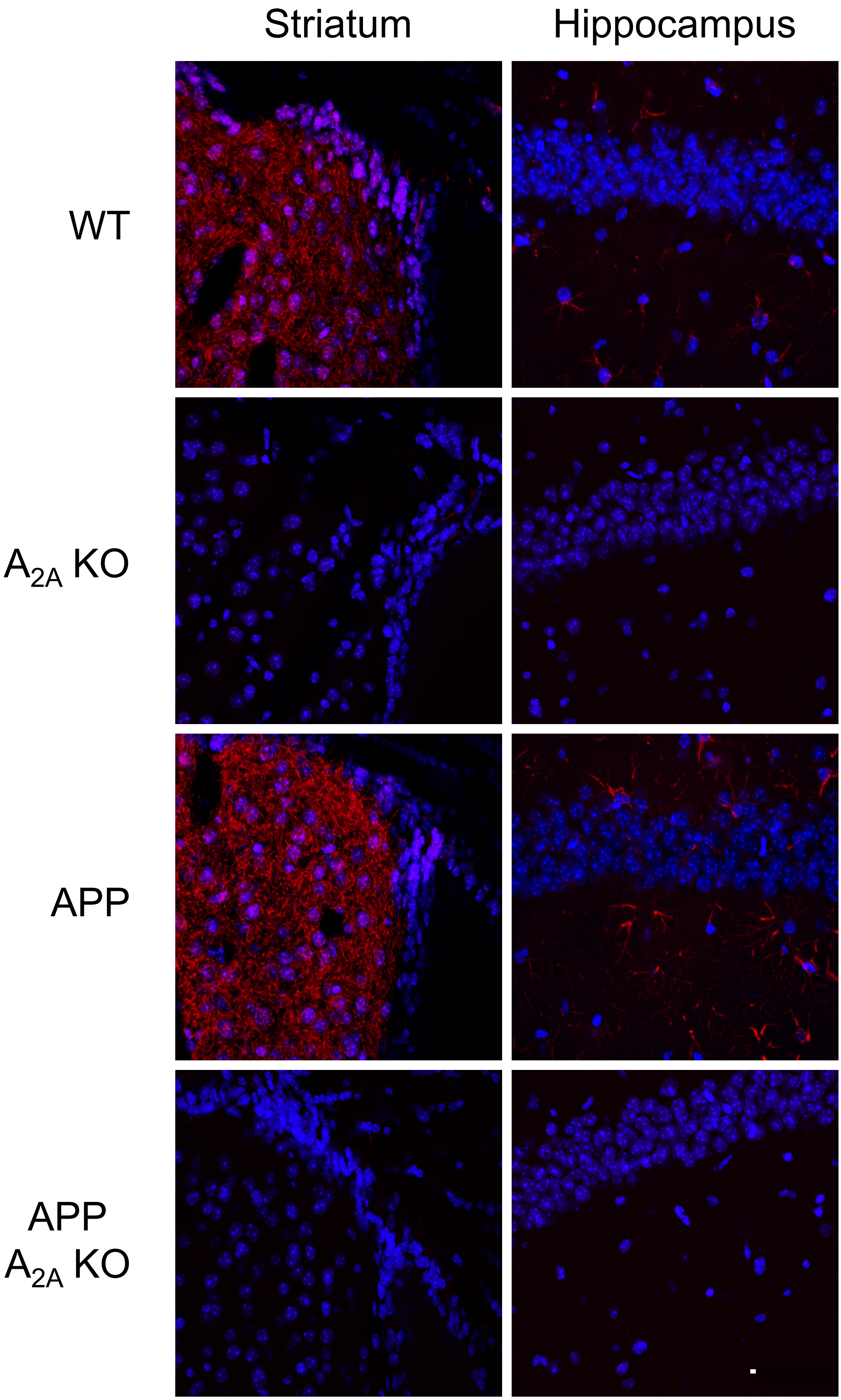

Supplementary Figure 2. A_{2A}R immunohistochemistry in the striatum (left) and hippocampus (right) of WT and APP/PS1 mice of 9-10m of age expressing (WT, APP/PS1) or not (A_{2A} KO; APP A_{2A} KO) A_{2A} receptors. Mice were obtained by a crossing of APP/PS1 A_{2A} +/- mice with A_{2A}R +/- mice. Results support the specificity of astrocytic expression of A_{2A}R in APP/PS1 mice. Scale bar = 50 μm.

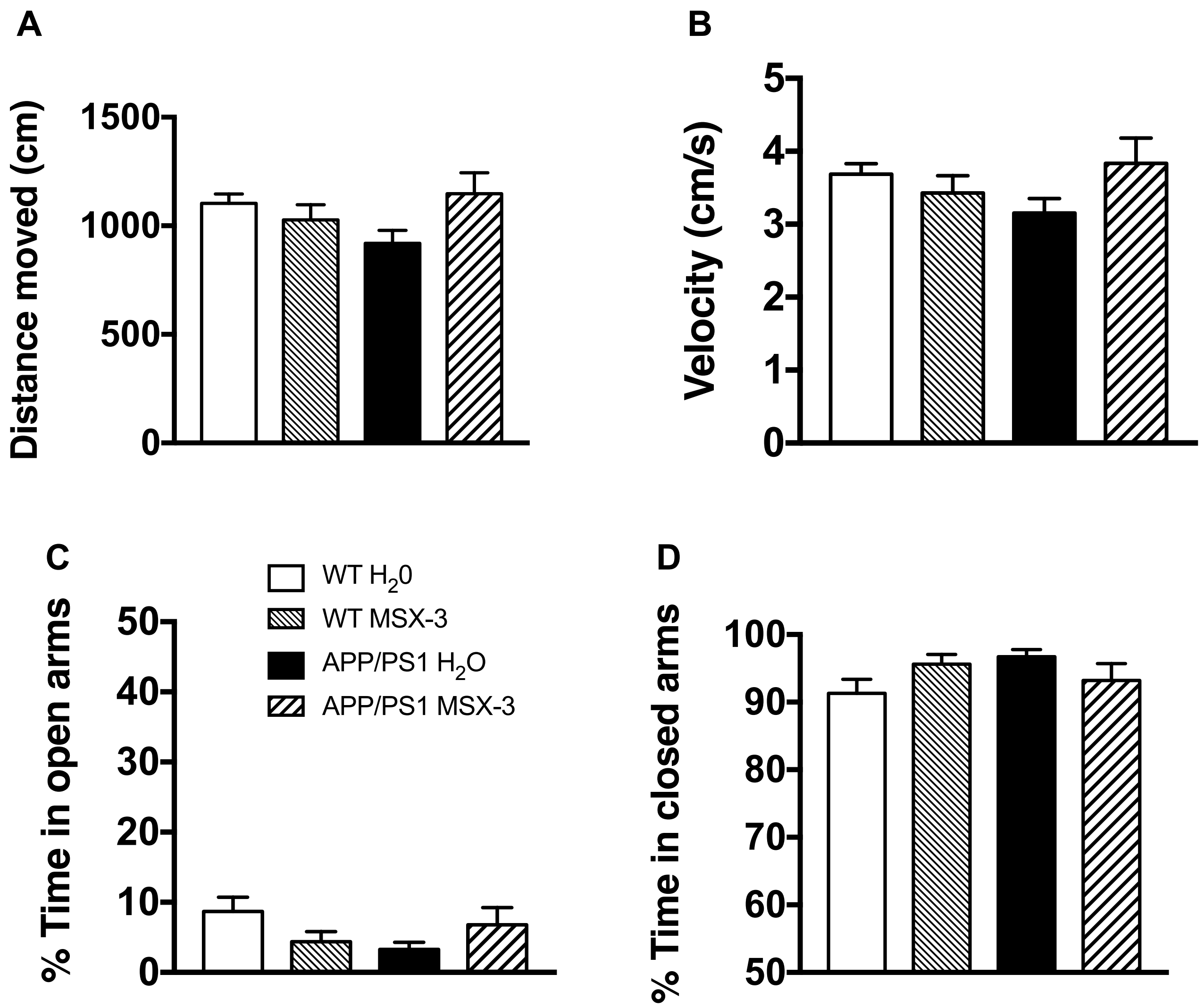

Supplementary Figure 3. Impact of MSX-3 on anxiety behaviour in WT and APP/PS1 mice. Anxiety levels were analysed using an elevated plus maze task and are reflected by the percentage of time spent in the open (C) and closed arms (D). None of them were affected by the treatment in WT and APP/PS1 animals. Neither distance moved (A) nor velocity (B) was significantly modified by the treatment.

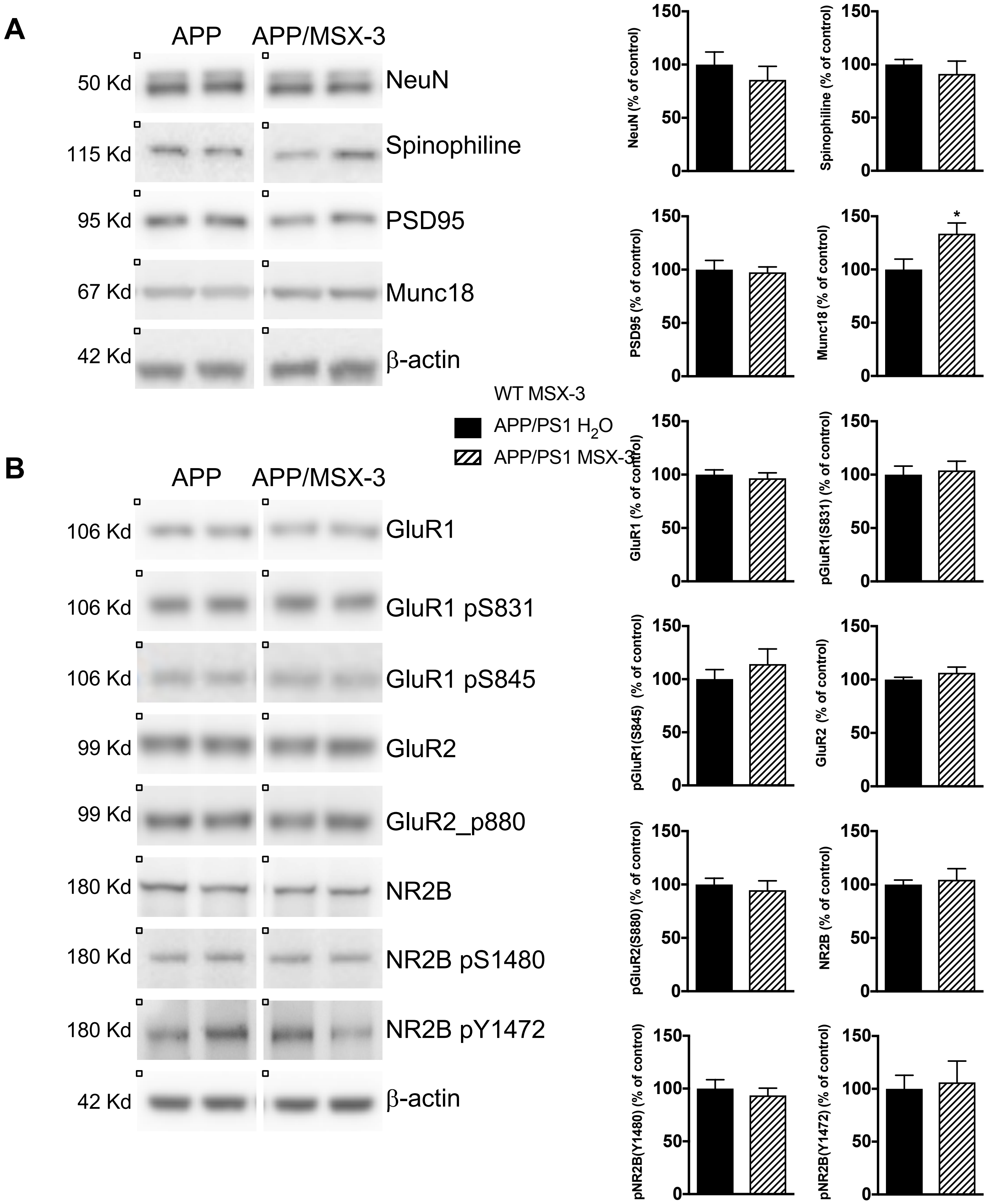

Supplementary Figure 4. Impact of MSX-3 on hippocampal synaptic markers in APP/PS1 mice. (A) MSX-3 treatment increased the levels of expression of the presynaptic marker Munc-18 in the hippocampus of APP/PS1 mice (*p<0.05, Student's t-test), while no effect was found regarding neuronal (NeuN) and postsynaptic markers (spinophilin and PSD95; p>0.05, Student's t-test). (B) No difference was found in the expression levels of markers involved in synaptic transmission and plasticity such as glutamatergic AMPA (GluR1, GluR2) and NMDA (NR2B) receptors and their phosphorylation (pGLUR1 at Serine 831 and 845, pGluR2 at Serine 880 and pNR2B at threonine 1480 and 1472; p>0,05, Student's t-test). N= 6 per group; Results are expressed as mean ± s.e.m.

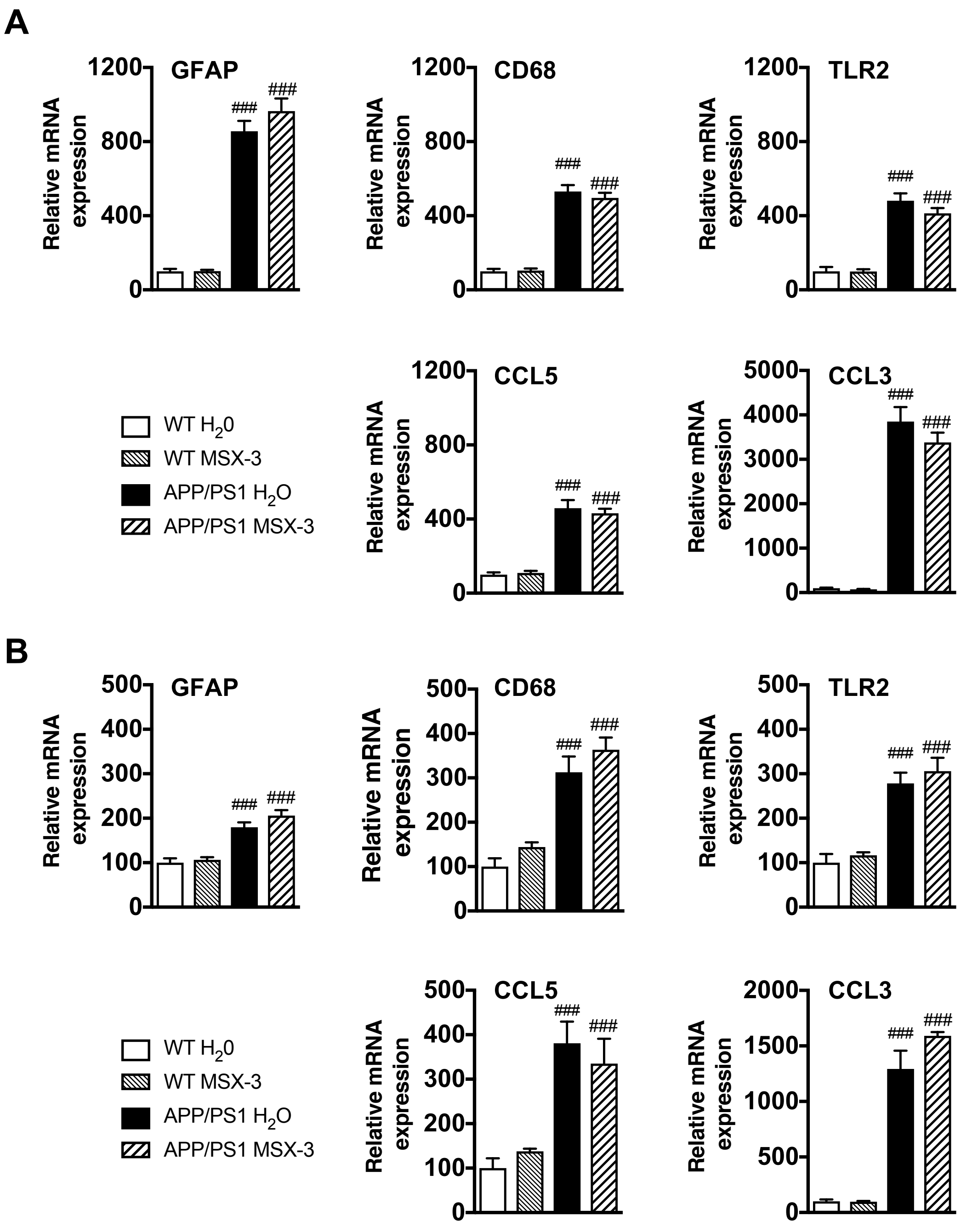

Supplementary Figure 5. Impact of MSX-3 on cortical and hippocampal neuroinflammatory markers in APP/PS1 mice. Quantitative PCR analysis indicate an significant upregulation of GFAP, CD68, TLR2, CCL5 and CCL3 in both the cerebral cortex (A) and the hippocampus (B) of APP/PS1 mice as compared to WT animals (###$p<0.001$ vs. WT H2O using One-way ANOVA followed by LSD Fisher post-hoc test). MSX-3 treatment had not effect in neither WT nor APP/PS1 mice. N=6 per group; Results are expressed as mean ± s.e.m.
